# Metabolic reprogramming in T cell senescence: a novel strategy for cancer immunotherapy

**DOI:** 10.1038/s41420-025-02468-y

**Published:** 2025-04-09

**Authors:** Li Liu, Zhanying Hao, Xi Yang, Yan Li, Siyang Wang, Linze Li

**Affiliations:** 1 The Operation Room, Shengjing Hospital of China Medical University, Shenyang, China; 2 Department of General Surgery, The Fourth Affiliated Hospital of China Medical University, Shenyang, China; 3 Department of General Surgery, Sanya People’s Hospital, Sanya, China; 4 Department of Anesthesiology, The Fourth Affiliated Hospital of China Medical University, Shenyang, China; 5 The Operation Room, The Fourth Affiliated Hospital of China Medical University, Shenyang, China

**Keywords:** Tumour immunology, Senescence

## Abstract

The complex interplay between cancer progression and immune senescence is critically influenced by metabolic reprogramming in T cells. As T cells age, especially within the tumor microenvironment, they undergo significant metabolic shifts that may hinder their proliferation and functionality. This manuscript reviews how metabolic alterations contribute to T cell senescence in cancer and discusses potential therapeutic strategies aimed at reversing these metabolic changes. We explore interventions such as mitochondrial enhancement, glycolytic inhibition, and lipid metabolism adjustments that could rejuvenate senescent T cells, potentially restoring their efficacy in tumor suppression. This review also focuses on the significance of metabolic interventions in T cells with aging and further explores the future direction of the metabolism-based cancer immunotherapy in senescent T cells.

## Facts

- Senescent T cells in tumors show increased glycolysis, lipid accumulation, and mitochondrial dysfunction;
- mTOR and p38 MAPK pathways drive metabolic changes that promote T cell senescence;
- Restoring mitochondrial function can rejuvenate senescent T cells and enhance anti-tumor immunity

## Open questions

- What are the key metabolic triggers that initiate T cell senescence in the tumor microenvironment?
- Can metabolic interventions selectively rejuvenate senescent T cells without impairing other immune subsets?
- How does the tumor microenvironment influence the metabolic fate of aging T cells?

## Introduction

T cell senescence is the process in which immune cells experience functional decline due to aging, playing a critical role in the development and progression of cancer [1]. As T cells age, they undergo significant metabolic reprogramming, which not only alters their energy production pathways but also results in reduced proliferative capacity and decreased cytokine production, thereby weakening their effective immune response to tumors [2]. This highlights the need for targeted therapies that can modify the metabolism of senescent T cells to restore their function and enhance tumor suppression [3].

The metabolic changes in senescent T cells are mainly characterized by increased glycolysis, altered lipid metabolism, and impaired mitochondrial function [4]. These metabolic changes are not just a natural consequence of aging; they also promote tumor immune evasion. Cancer cells promote T cell senescence by altering T cell metabolic pathways, further driving tumor progression, highlighting the complex link between metabolism, senescence, and cancer progression [5].

This perspective identifies certain features of altered metabolism of senescent T cells inside the tumor microenvironment can be considered as a potential therapeutic target [6]. Strategies that can reverse these metabolic shifts or prevent their occurrence have the potential to restore the functionality of senescent T cells. This not only aids in effective tumor surveillance but also enhances the response to cancer immunotherapy [7]. Thus, this review will discuss the current knowledge of metabolic reprogramming of senescent T cells in regard to cancer, how these changes implicate T cell dysfunction as well as cancer progression. It will also delve into emerging therapeutic strategies aimed at targeting these metabolic alterations, with a focus on rejuvenating the anti-tumor immunity of senescent T cells. This approach represents a novel frontier in the fight against cancer, offering hope for more effective treatments that leverage the body’s own immune system.

## Markers of T cell senescence

Aging leads to the accumulation of dysfunctional, terminally differentiated T cells. Chronic inflammatory stimuli drive these cells to adopt senescent or exhausted phenotypes, which impair their immune functions. Both types of cells exhibit deficiencies in tumor immunology, with distinct molecular and functional profiles [8].

**Fig. 1:**
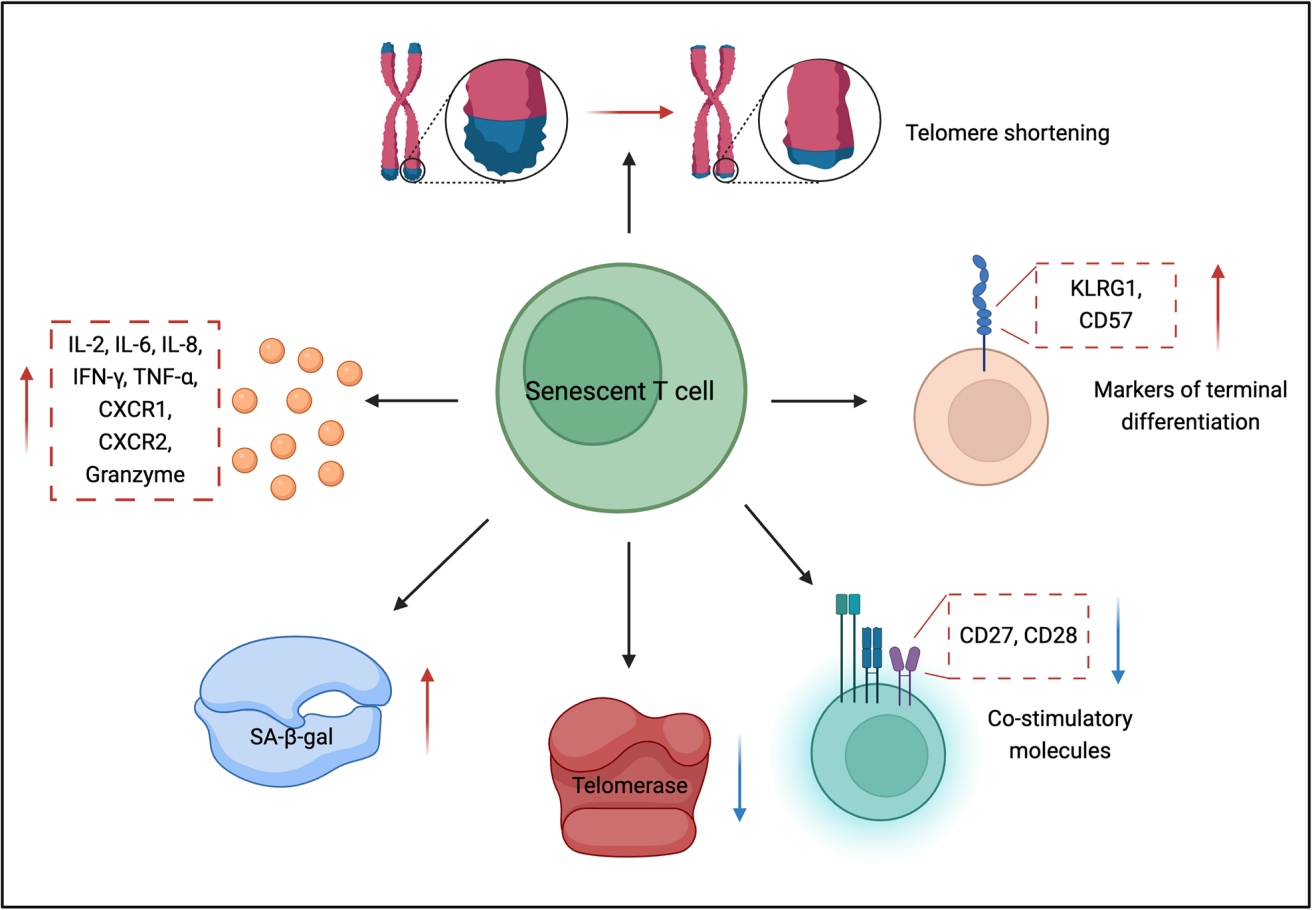
Markers of T cell senescence.

Senescent T cells, upon stimulation, adopt a senescence-associated secretory phenotype (SASP), secreting various inflammatory proteins such as IL-2, IL-6, IL-8, interferon-γ (IFN-γ), TNF-α, and chemokine receptors CXCR-1 & CXCR-2, although they do not divide [9, 10]. In contrast, exhausted T cells show reduced production of IL-2, TNF-α, and other inflammatory factors, alongside a decline in cell division, eventually losing effector capabilities. This highlights the distinct nature of senescence and exhaustion [11] (Fig. 1). Senescent T cells also show typical signs of aging, including shorter telomeres, DNA damage markers such as γH2AX foci, resistance to apoptosis, and age-related β-galactosidase (SA-β-gal) activity. They lose co-stimulatory markers like CD27 and CD28, while expressing higher levels of terminal differentiation markers such as KLRG1 [12]. CD57, typically associated with active proliferation in vivo, is also a marker of senescent T cells [13].

Moreover, the susceptibility to aging differs between CD4+ and CD8+ T cells, with CD8+ T cells developing an immunosenescence phenotype more rapidly. Both CD4+ and CD8+ TEMRA cells undergo similar aging changes, but CD8+ TEMRA cells accumulate more quickly, likely due to metabolic differences [12, 14]. Compared to their CD8+ counterparts, CD4+ TEMRA cells maintain healthier mitochondria, while CD8+ cells are more prone to mitochondrial degradation [15]. Additionally, senescent T cells downregulate TCR signaling while upregulating the expression of the antagonistic NK receptor NKG2D and the NK adapter molecules DAP10 and DAP12, which enhance cytotoxicity against cells expressing NKG2D ligands [16]. Furthermore, Mogilenko et al. described both isolated and common immune changes, and identified a new subpopulation of age-related CD8+ T cells that express granzyme K, known as Taa cells. These Taa cells are highly clonal and possess distinct epigenetic and transcriptional profiles. They arise due to AIDS, and in tissues such as skin, they exhibit signs of exhaustion and homing, indicating that they belong to the final stages of the immunological aging process [17] (Fig. 1).

## Metabolic changes in senescent T cells in cancer

The metabolic changes in senescent T cells are closely linked to the specific markers of senescence. These metabolic shifts, including increased glycolysis, altered lipid metabolism, and impaired mitochondrial function, contribute to the accumulation of senescence-associated markers. For example, the increased glycolysis commonly seen in senescent T cells leads to the secretion of pro-inflammatory cytokines, which in turn promote the expression of markers such as SA-β-gal and CD57 [18]. These metabolic changes not only affect T cell function but also play a significant role in the formation and maintenance of the senescent phenotype. This section will explore these metabolic changes in senescent T cells in cancer, examining how these alterations drive T cell dysfunction and contribute to immune evasion by tumors.

### Alterations in glucose metabolism

**Fig. 2:**
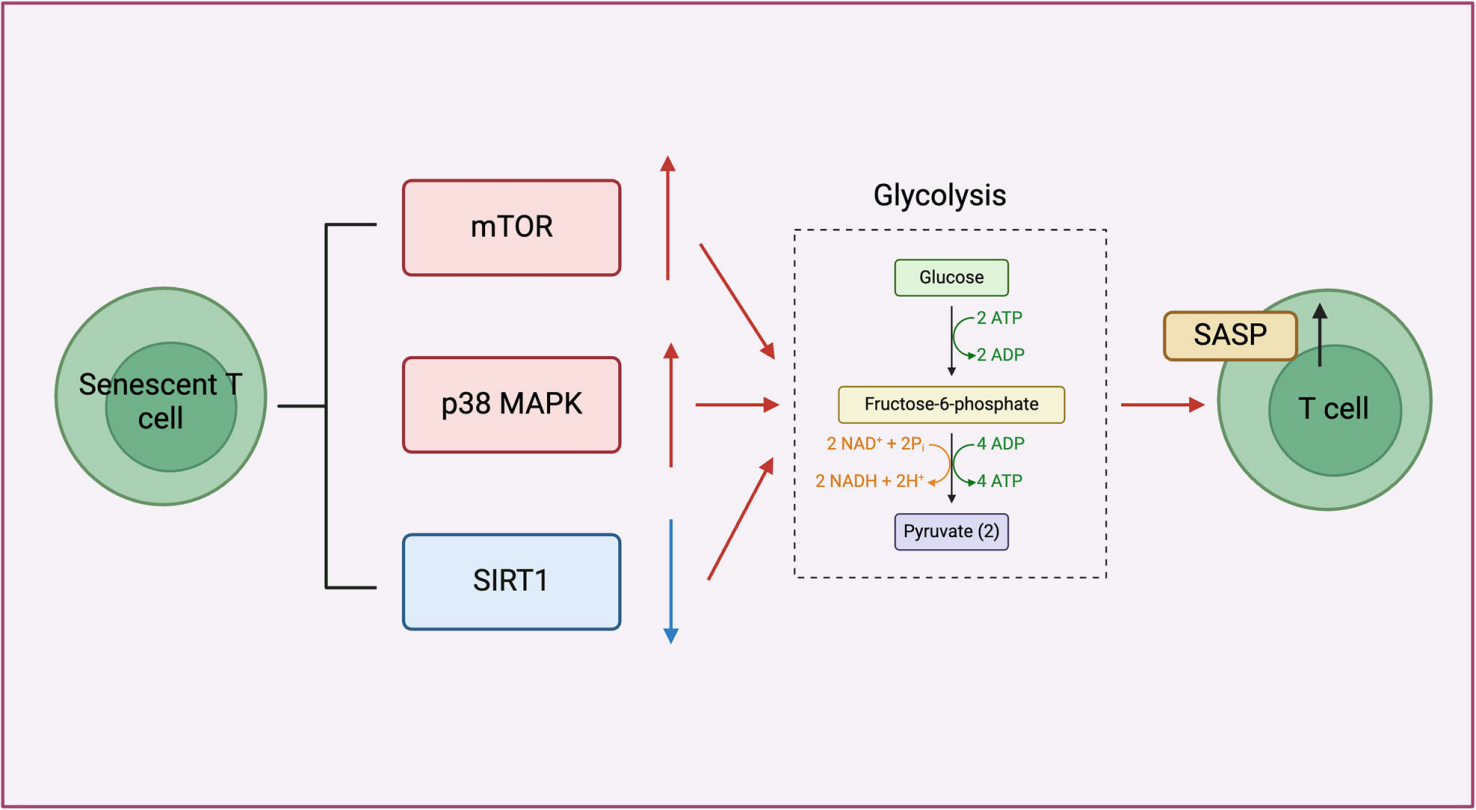
Alterations in glucose metabolism in senescent T cells.

As age increases, the accumulation of aging T cells, like other types of aging cells, exhibits increased glycolytic metabolism in a steady state. This, in turn, ultimately promotes effector differentiation and inflammation of stimulated T cells [19–21]. Similarly, tumor-induced senescent T cells show high glycolytic metabolism characteristics. Liu et al. observed that inhibition of glycolytic pathway of senescent CD8+ T cells due to breast cancer cells also decreases the secretion of SASP. Additionally, qPCR results show upregulation of glycolysis-related enzyme expression in senescent CD8+ T cells [22]. As a result, the knowledge of the molecular control of glycolytic activation in aging T cells enhances the current perspectives on elements associated with molecular aging of T cells and contributes to the identification of novel approaches to address the issue (Fig. 2).

One should mention that using the model of aging T cells, activation of the mammalian target of rapamycin (mTOR) and p38 MAPK signaling, leading to the formation of the SASP [23–25]. Importantly, in the analysis of lymphoma patients, it was found that there is enhanced PI3K/AKT/mTOR signaling in patient CD8+ T cells, which promotes aerobic glycolytic metabolism in these cells, driving the senescence of CD8+ T cells [26]. However, current research has not yet intervened in mTOR signaling in tumor-induced senescent T cells and observed changes in glycolysis levels. This will be an area that needs further refinement in the future. Notably, activation of the mTOR signaling pathway also occurs in aging CD4+ T cells, and this activation is mediated by the upregulation of miR-21 in CD4+ T cells of elderly individuals [27]. However, the study did not clarify whether it relies on the activation of mTOR signaling to promote glycolytic metabolism and induce the senescence of CD4+ T cells. Importantly, in aging CD4+ T cells induced by tumor-derived Tregs, high concentrations of glucose intervention can inhibit CD4+ T cells senescence [28]. The above study results further support the scientific hypothesis that enhanced glycolytic metabolism induced by the mTOR signaling pathway helps drive the aging of CD4+ T cells, becoming a potential research direction in cancer in the future. Interestingly, research has revealed that Tregs, a subtype of CD4+ T cells, show enhanced glycolytic metabolism induced by the mTOR signaling pathway. Moreover, TLR8 signaling can suppress this metabolic activation, thereby bolstering anti-tumor immunity in vivo in cases of melanoma [28]. Given that the aging of Tregs can reduce their immunosuppressive capacity [29], it raises the intriguing possibility that enhanced glycolytic metabolism, driven by the mTOR pathway, might amplify anti-tumor immunity by promoting the senescence of Tregs. To explore this hypothesis further, future studies could design experiments to specifically activate the mTOR signaling pathway in Tregs. Researchers could use both pharmacological activators of mTOR and genetic modifications to upregulate the pathway selectively in Tregs. Subsequent experiments could measure changes in glycolytic metabolism by assessing key metabolic markers such as glucose uptake and lactate production. Additionally, the expression of aging markers, like β-galactosidase, in Tregs could be analyzed to determine the extent of senescence induced by mTOR-driven metabolic changes. Regarding p38 MAPK signaling, its activation in tumor cells has been shown to enhance glycolysis [30, 31]. Since p38 MAPK signaling is known to induce aging in T cells [32], it is intriguing to consider the potential role of glycolysis enhancement mediated by this pathway in driving T cell aging. Moreover, an increasing number of studies have linked human diseases and normal physiological processes to epigenetic changes. Early studies demonstrated that the level of DNA hydroxymethylation in peripheral blood T cells from 53 human volunteers decreases with age, suggesting the presence of novel epigenetic regulatory mechanisms in aging T cells [33]. Notably, findings showed that the MAPK signaling pathway becomes significantly hypermethylated with age, and is therefore likely silenced at the epigenetic level in aging naive CD4+ T cells [23]. This suggests that age-associated DNA methylation may influence T cell aging by mediating changes in T cell glycolysis. Future research could focus on delineating the mechanistic links between p38 MAPK activation and metabolic changes in T cells. Specifically, studies could examine how enhanced glycolysis affects the aging process at the molecular level, potentially contributing to immunosenescence. One promising direction is to use both in vitro and in vivo models to explore the effects of modulating p38 MAPK signaling on T cell metabolism and longevity. Researchers could investigate whether inhibiting or enhancing p38 MAPK signaling alters the metabolic profile of T cells and assesses the subsequent impact on their function and lifespan. Additionally, investigating the interplay between p38 MAPK signaling and other aging-related pathways, such as sirtuins and mTOR, could provide deeper insights into the comprehensive network of signals that govern T cell aging.

The transformation of aging T cells into a predominantly glycolytic metabolic state is closely linked with their more differentiated or “unrestrained” phenotype. Thus, a vast decrease in Sirtuin 1 (SIRT1) expression is identified in aging people within the body tissues, a phenomenon that contributes to organ aging and the progression of senescence [34, 35]. Crucially, in aging CD8+CD28- T cells, the downregulation of SIRT1 results in enhanced proteasomal degradation of FoxO1, a key SIRT1 target involved in the transcriptional reprogramming of these cells. This degradation leads to an increased glycolytic capacity under resting conditions and elevated secretion of granzyme B [19]. Similarly, in CD4+ T cells, the suppression of SIRT1 expression can trigger an aging phenotype, defined by the losing of the telomere length and the gaining of β-galactosidase [36, 37]. Notably, in a melanoma animal model, CD4+ helper T cells 9 (Th9) that lack SIRT1 show an increased glycolytic metabolism. Intriguingly, SIRT1 regulates the glycolysis in Th9 cells through targeting the mTOR/HIF1α signaling pathway [38]. Altogether, these studies indicate that in CD4+ T cells infiltrating tumor tissues, the absence of SIRT1 may drive cellular aging by enhancing glycolysis. However, there is a lack of direct aging phenotypes to validate this hypothesis, which could be a future research direction in various types of cancer.

### Alterations in lipid metabolism

**Fig. 3:**
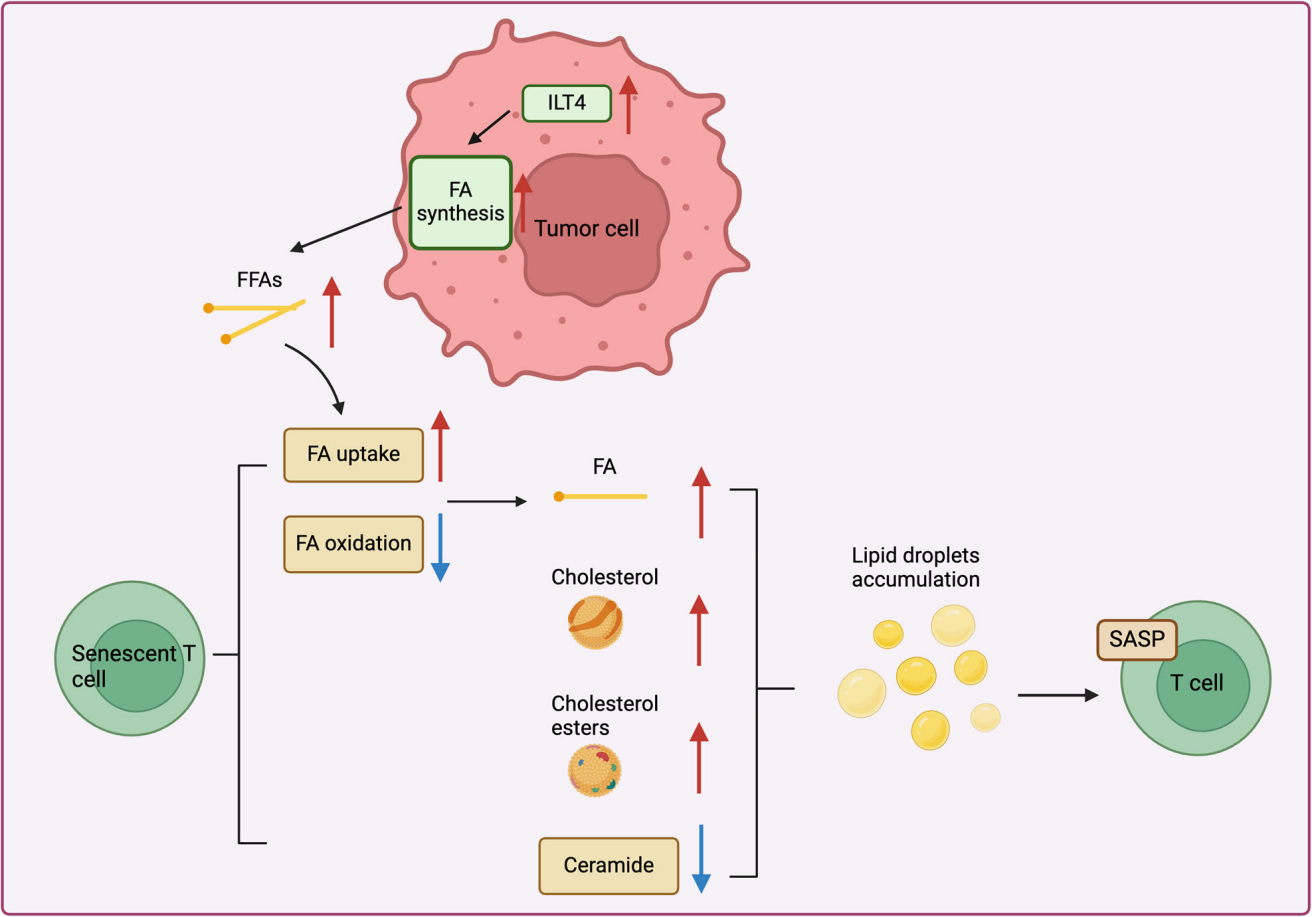
Alterations in lipid metabolism in senescent T cells.

Furthermore, lipids are an important energy source, can function as signaling molecules and regulate inflammation which is vital for proper T cells’ function in the tumor context [39]. Thus, lipid metabolism is also an essential factor in the rewriting of the metabolic profile of T cells during their state of senescence in cancer (Fig. 3).

Research has found that compared to middle-aged individuals, elderly individuals have CD8+ T cells with higher levels of fatty acid (FA) uptake and lipid storage [40]. Further, the aging CD8+ T cell study in culture indicates that in aging CD8+ T cells uptake of fatty acids is augmented but there is defect in fatty acid oxidation (FAO), resulting in heightened lipid droplets (LDs) accumulation [41]. Interestingly, the present study established that breast cancer cells promote the formation of LDs within T cells, CD8 and CD4, which is responsible for T cell senescence. Further research has found that the MAPK and STAT signaling pathways upregulate the expression of cytosolic phospholipase A2 alpha (cPLA2α), promoting the formation of LDs and thereby inducing T cell senescence [22]. Importantly, lipidomic analysis results show a significant increase in cholesterol, cholesterol esters, and free fatty acids (FFA) in tumor-induced senescent T cells, all of which are key components of LDs. However, the levels of ceramide in senescent T cells are significantly downregulated [22].

Previous research indicates that T cells from elderly individuals accumulate higher cholesterol levels in their cell membranes compared to those from younger individuals [42]. Recent findings have further elucidated that deficiencies in ABCA1/ABCG1 contribute to an aging phenotype in T cells from middle-aged LDLR−/− mice. Specifically, this phenotype is marked by a six-fold increase in p21 mRNA expression in CD4+ T cells and a 2.5-fold increase in CD8+ T cells. Notably, the absence of ABCA1/ABCG1 enhances cholesterol accumulation (both free cholesterol and cholesteryl esters) in T cells, resulting in fewer naïve T cells being recruited from the periphery while and preserving thymocyte numbers [43]. Overall, it is evident that cholesterol build-up drives the aging phenotype in T cells. Regrettably, the effect of cholesterol on the responsiveness and related functions of CD3/CD8 after antigen stimulation is still not well understood.

Recent studies have discovered that long-chain acylcarnitine (LCAC), a compound formed by the esterification of long-chain fatty acids and carnitine, abnormally accumulates in hepatocellular carcinoma (HCC) tissues. Significantly, this accumulation is also noted in NKT cells infiltrating HCC, likely due to the increased expression of CD36, which recognizes and binds long-chain fatty acids (LAFAs), thus facilitating their entry into the cells. Moreover, the abnormal buildup of LCAC in NKT cells contributes to their senescence [44]. However, whether the senescence of NKT cells induced by LCAC is directly caused by LCAC itself or due to the accumulation of LAFAs resulting from LCAC conversion in NKT cells requires further investigation. Additionally, research by Gao et al. suggests that ILT4 in breast cancer and melanoma cells stimulates T cell senescence through enhancing fatty acid synthesis and lipid content in the cancer cells [45]. Notably, lipid accumulation in the tumor microenvironment increases fatty acid uptake by T cells [46]. Thus, ILT4-mediated upregulation of FA levels in the tumor microenvironment may lead to abnormal FA accumulation in T cells, thereby driving T cell senescence [45].

Interestingly, Vaena and colleagues discovered that aging T cells isolated from aged mice exhibited an increased accumulation of C14/C16 ceramide in their mitochondria, inducing mitochondrial dysfunction and accelerating senescence. This specific ceramide-dependent aging process further diminished the T cells’ anti-tumor capacity against melanoma [47]. However, the lipidomic analysis done by Liu et al. on tumor-induced senescent T cells depicted a lower level of ceramides than regular cells. However, exogenous supplementation of ceramide significantly reversed the aging of T cells [22]. This highlights intriguing contradictions in observed phenomena. Notably, the chain length of ceramides plays a crucial role in their biological activity and effects, with different chain lengths exhibiting distinct functions and effects within cells [48]. This might explain why overall ceramide levels decrease in aging T cells, yet the levels of specific C14/C16 ceramides are upregulated. Future research should further analyze which ceramides are downregulated and identify the related molecular mechanisms. This information will aid in developing more precise targeted regulatory strategies. Furthermore, the process of T cell senescence caused by aging is considered to be different from that caused by tumors, while tumor cells may influence lipid metabolism of T cells by releasing extracellular factors.

Additionally, it is worth further exploration that dietary phospholipids have been shown to accelerate T cell aging in mouse models [49]. Conversely, another study indicated that in tumor-induced senescent T cells, phospholipid levels are decreased, and external supplementation of phospholipids reverses the aging of T cells [22]. Understanding the molecular mechanisms involved will provide new targets for cancer treatment in the elderly.

In summary, the studies mentioned above demonstrate that there is a significant imbalance in lipid metabolism during the aging process of T cells induced by tumors. However, the specific mechanisms causing this imbalance in lipid metabolism have not yet been fully elucidated, and the molecular mechanisms by which lipids regulate cellular senescence in T cells also require further exploration. Future research needs to delve deeper into the key regulatory factors of lipid metabolic pathways and how these factors affect the functionality and lifespan of T cells. This endeavor will not only help in identifying the molecular changes that occur during the aging of T cells but may possibly outline new strategies that may be useful in anticancer therapies. That is why identifying the actions of lipid metabolism for T cell aging is important to set up immune outcomes in cancer treatment.

### Metabolic changes based on mitochondria

**Fig. 4:**
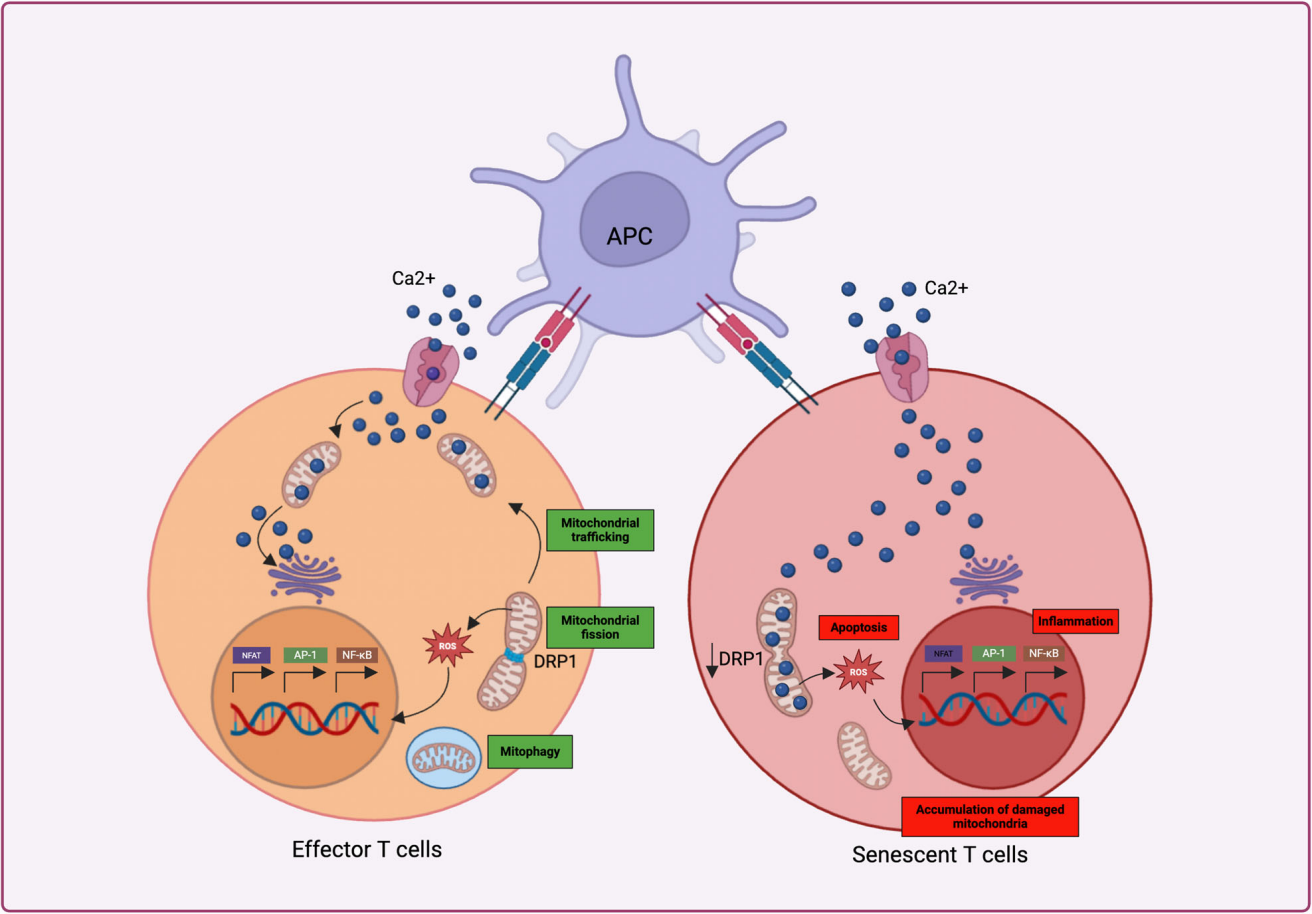
Mitochondrial dysfunction consequences in senescent T cells.

Mitochondria play crucial roles in energy generation [50], signal transduction [51], cell death [52], and the aging process [1] within cells. As the age increases, there is a decline in the efficiency of mitochondria and increase in the leakage of reactive oxygen species (ROS) from the mitochondria. This process is widely recognized as both a fundamental cause and a direct consequence of aging. During the aging process, the fidelity of respiratory chain complex assembly diminishes, resulting in inefficient electron transport chain (ETC) function and a consequent imbalance in redox homeostasis. This imbalance subsequently affects changes in mitochondrial membrane potential (MMP) [53]. Complexes I, II, and III are the primary producers of ROS in cells, and their stability has been shown to decrease with age [54, 55]. Excessive ROS production via complexes I and III can be mitigated by their assembly into supercomplexes. These supercomplexes where complexes I, III, and IV interact increase the activity of complex I also the efficiency of the electron transport, reducing the risk of electron leakage and, consequently, lowering ROS production. However, as age increases, the stability of the ETC supercomplexes declines, resulting in oxidative stress through the generation of more ROS and increased load on the mitochondria hence causing mitochondrial damage [56, 57]. In senescent CD8+ T cells, mitochondria are enlarged and exhibit hyperpolarized membranes, which lead to an increased production of ROS, contributing to mitochondrial dysfunction [58]. In tumor-infiltrating CD8+ T cells, there is a notable increase in mitochondrial ROS levels, likely due to hyperpolarized mitochondria. Consequently, this elevation in ROS is associated with enhanced DNA damage within these cells [59]. This implies that the tumor microenvironment could influence mitochondrial oxidative stress in CD8+ T cells, leading to their senescence and inhibiting their ability to fight cancerous cells. Notably, when evaluating a subset of CD8+ T cells, it has been found that Tc9 cells are longer living than Tc1 cells in tumor site. This difference is attributed to lower levels of mitochondrial ROS, which are produced by lipid peroxidation in Tc9 cells. This finding further suggests that lower mitochondrial ROS levels contribute to delaying the aging of CD8+ T cells [60]. Similarly, elevated mitochondrial ROS levels in CD4+ T cells induce mitochondrial dysfunction, precipitating premature senescent of T cells through dysregulation of superoxide dismutase 1 (SOD1) and apurinic/apyrimidinic endonuclease 1 (APE1) [61] (Fig. 4).

Mitochondria are dynamic structures that adjust their size, shape, and intracellular location through processes of fusion and fission, adapting to meet their metabolic needs and functional roles [62]. These processes, collectively known as mitochondrial dynamics, are crucial for mitochondrial metabolism and quality control. As aging progresses, indicating that mitochondrial dynamics are dysregulated, resulting in the formation of aberrant mitochondria [63, 64]. The process of mitochondrial fission involves proteins such as Fission 1 Homolog (FIS1), Mitochondrial Fission Factor (MFF), Mitochondrial Dynamics Protein of 49 kDa (MID49), and Mitochondrial Dynamics Protein of 51 kDa/Mitochondrial Elongation Factor 1 (MID51/MIEF1). These proteins recruit the key fission protein, Dynamin-Related Protein 1 (DRP1), to the fission sites to constrict and facilitate the division of mitochondria [65]. This fission process increases the production of ROS, accelerates cell proliferation, and mediates apoptosis [66–68]. In contrast, mitochondrial fusion is initially mediated by mitofusins 1 and 2 (MFN1 and MFN2), which catalyze the fusion of the outer mitochondrial membrane, then by the Optic Atrophy 1 (OPA1) protein which functions as pro-fusion protein of IMM to form large mitochondria [69]. Fused mitochondria can buffer localized mitochondrial damage and mtDNA mutations [70], maximize oxidative phosphorylation (OXPHOS) activity [71], and enhance interactions with the endoplasmic reticulum (ER) [72]. Interestingly, a single-cell RNA sequencing study revealed that elderly people have higher T cell FIS1, MFF, and DRP1 levels compared to young individuals. Inhibition of MitoSTAT3 limits DRP1 while boosting MFN1 expression, aiding in the restoration of mitochondrial morphology to that observed in younger T cells [73]. This indicates that mitochondrial fusion could be used as the stress resulting from aging or senescence. Similarly, in the aging process, ceramide accumulation induced by aging stress inhibits protein kinase A (PKA), thereby removing PKA’s inhibition of phosphorylation at the DRP1 S637 site, which in turn leads to the activation of DRP1 in T cells. This results in the restriction of longevity and immune suppress capacity of the older T cells and decreases their capacity to affect melanoma [47]. Importantly, the activation of DRP1 induces mitochondrial fission in senescent T cells, leading to dysfunction in mitochondrial dynamics [47]. In light of these data, changes in mitochondrial dynamics might affect the decrease in function of senescent T cells infiltrating the tumor. However, whether alterations in the dynamic structure of mitochondria can drive the aging of T cells, thereby diminishing their anti-tumor immune capabilities, remains a question that requires further investigation. Future research could focus on strategies for targeted intervention in mitochondrial fission or fusion proteins in T cells, while also exploring the upstream and downstream factors and signaling pathways that interact with key proteins such as DRP1, FIS1, MFN1, MFN2, and OPA1.

## Therapeutic strategies for targeting metabolic reprogramming in senescent T cell

**Fig. 5:**
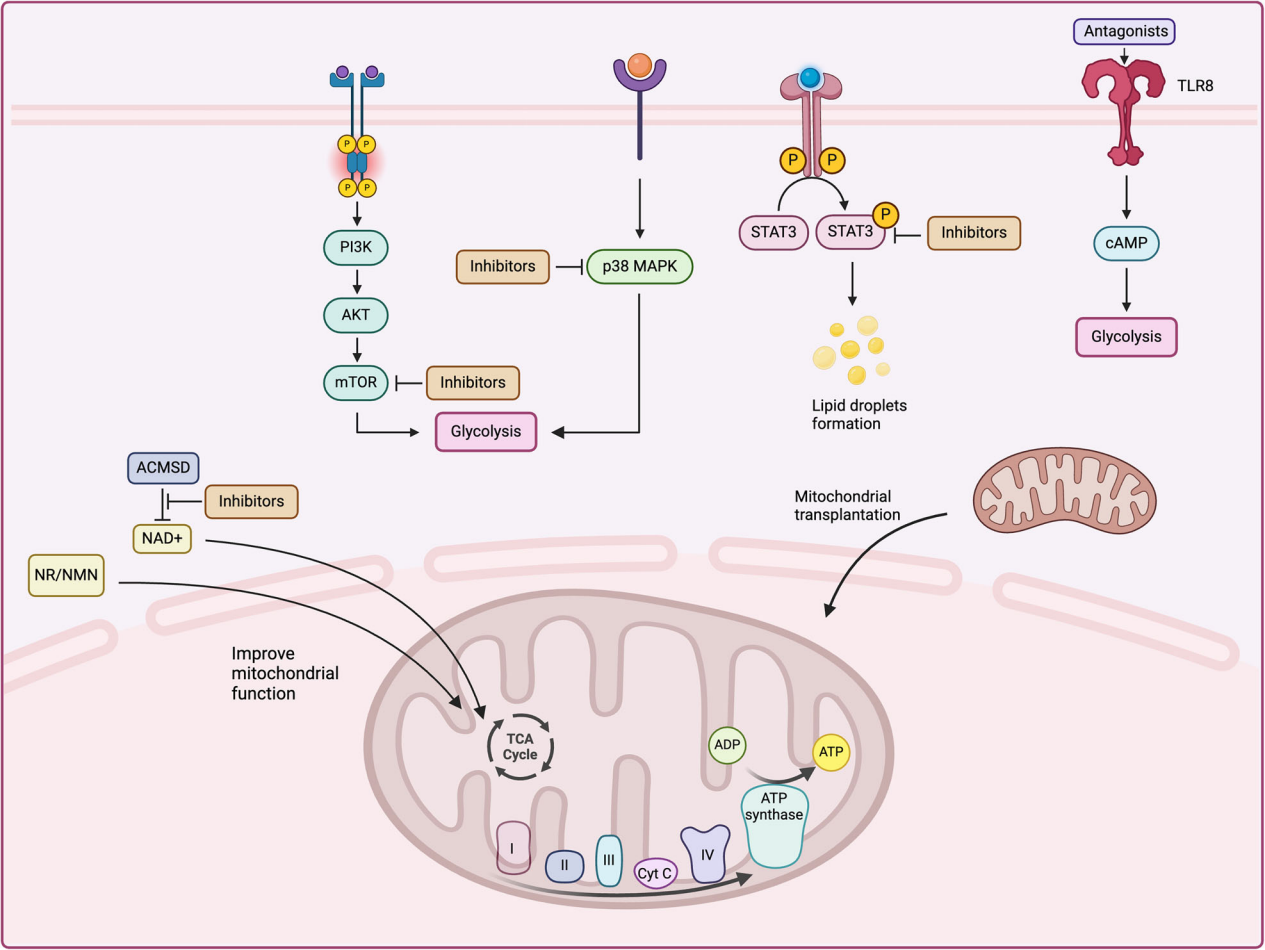
Therapeutic strategies for targeting metabolic reprogramming in senescent T cell.

Therefore, in the perspective of immunology and aging the defective T cell functions are revealed to be due to the presence of senescent cells. These cells’ metabolic changes are associated with their new functions and decreased efficiency in identifying and eliminating pathogens and tumors. Therefore, understanding and targeting the metabolic pathways in senescent T cells offers a promising avenue to rejuvenate these crucial components of the immune system and restore their vitality. This section delves into the specific strategies that can modify the metabolic environment of senescent T cells, aiming to reverse their senescent state and enhance their immune functions (Fig. 5).

### Targeting metabolic pathways in senescent T cells

Thus, the critical metabolic signaling pathways that regulate the induction of T cell senescence represent a key biological target for effectively enhancing anti-tumor immunity. Additionally, fibroblasts have demonstrated the ability to bypass growth arrest by suppressing cyclin-dependent kinases involved in early senescence [74, 75]. This finding therefore provides useful pointers that may help explain how T cell senescence might be reversed.

Increased mTOR activity is known to be involved in T cell aging, making it a potential therapeutic target. It is crucial to note that treatment with rapamycin suppresses mTOR activity within the body, inhibiting glycolysis in aging T cells. It returns some naive T cells slightly, which significantly reduces T cell abnormalities in this in vitro case [26]. Moreover, the in vivo analysis of accelerated aging carried out using rapamycin show that it improves the numbers of CD8+ T cells. This suggests that targeting the mTOR signaling pathway (by inhibiting glycolysis) is a viable approach to mitigating T cell senescence [26]. Notably, mTOR inhibitors have been clinically applied and have shown promising results. Everolimus, for instance, has been found to beneficially impact immune senescence, particularly T cell aging, in elderly volunteers [76], and this drug has been safely used in treating age-related diseases (NCT02874924) [76]. Notably, everolimus has been demonstrated to revert immune dysfunctions in numerous neoplastic diseases, such as breast and prostate cancer and overall result in favorable patient prognosis [77, 78]. This underscores the potential of mTOR inhibitors not only in managing immune aging but also as therapeutic agents in oncology. However, mTOR inhibitors, such as rapamycin and everolimus, have been shown to cause several side effects when used for extended periods. These include impaired tissue regeneration, reduced immune response, and increased risk of infection, which could complicate their use, especially in older populations with preexisting conditions [79]. Moreover, there are off-target effects, such as changes in metabolic regulation that may affect non-immune cells or other aspects of immune function that are not related to aging. For example, chronic use of mTOR inhibitors can affect muscle regeneration and bone density, limiting their clinical applicability [80]. Therefore, a more refined dosing approach, alongside targeted delivery systems, is needed to reduce these risks and maximize the therapeutic benefits of mTOR inhibition.

Likewise, p38MAPK is another critical target responsible for counteracting T cells senescence. Therefore, the p38MAPK inhibitors are valuable in developing an effective strategy to address T cell senescence resulting from DNA damage. The efficacy and safety of these inhibitors have already been established in clinical settings, particularly in treating complex conditions like myelodysplastic syndrome and multiple myeloma (MM) [81–83]. Further research underscores that targeting the MAPK signaling pathway can revitalize essential cellular processes, enabling T cells to regain their cell cycle activity and proliferative capabilities [24]. Moreover, MAPK inhibitors have gained considerable traction in oncology, especially in the treatment of melanoma. It has also been established that usage of their products enhances dramatically the capacity of the immune system to detect and destroy melanoma cells, effectively enhancing T cell-mediated tumor immunity. Importantly, these benefits are achieved without compromising the overall functionality of lymphocytes, ensuring that the immune system’s integrity and response capacity are maintained. This dual benefit of MAPK inhibition not only bolsters anti-tumor activity but also preserves the health and functionality of the immune system, making it a promising avenue for broader immunotherapeutic applications [84, 85]. While p38MAPK inhibitors show great promise, they are not without their challenges. Off-target effects associated with the inhibition of p38MAPK may result in the suppression of pro-inflammatory responses that are crucial for immune surveillance, potentially leading to an increased susceptibility to infections [86]. Therefore, prolonged inhibition of this pathway might lead to adverse impacts on the overall health of the immune system. Research is ongoing to identify specific p38MAPK inhibitors that selectively target the aging T cell population without impacting other immune or non-immune cells.

Furthermore, the aforementioned studies indicate that the STAT signaling pathway is involved in the formation of LDs and the induction of mitochondrial dynamics dysfunction, factors that drive the aging of T cells. Therefore, modulating and especially inhibiting STAT signaling may also counteract T cell exhaustion and increase anti-tumor immunity. The other is the stimulation of the TLR8 signaling in both Treg and tumor cells. cAMP inhibitors can avoid tumor-induced T cell senescence by rejuvenatingTLR8 signaling [87]. TLR8 antagonists decrease cAMP levels in tumor cells, and inhibit glycolysis in tumor-derived Tregs, without disrupting the metabolism of effector T cells [88]. This strategy is quite effective in preventing T cell from becoming senescent. The targeting of STAT signaling and TLR8 signaling provides a novel avenue for enhancing anti-tumor immunity. However, these pathways are involved in many aspects of immune cell function, and the inhibition of STAT or TLR8 could potentially affect other immune cell types or immune responses. As such, the selectivity of these interventions is critical to avoid non-specific effects on other immune cells, such as dendritic cells [89, 90] or NK cells [91, 92], which also play pivotal roles in immune responses. More research is needed to ensure that modulating these pathways will not lead to unintended immune suppression or dysfunction.

### Enhancing mitochondrial function

It is suggested that T cells become senescent due to decrease in metabolic activity and impaired function of the mitochondria. Mitochondria are pivotal in cellular iron utilization and metabolism, playing as a critical role as the cofactor where various mitochondrial proteins, such as iron-sulfur clusters and heme-containing proteins, are synthesized. Jovian et al. cultured cells under growth medium containing different concentrations of FeSO4 and performed growth assays at various time points (16, 24, and 48 h). They noted that cell division attained its maximum after 24 h of treatment with FeSO4 and the chronological lifespan (CLS) of the cells was then measured. According to the observed results, various concentrations of FeSO4 increased the CLS of yeast. Similarly, the study evaluated various concentrations of FeCl3, yielding comparable outcomes to those with FeSO4. The following experiments show that iron supplementation can slow the aging process and increase lifespan due to the enhancement of the functioning of mitochondria [93]. In addition, it has also been established that other T cells which have a damaged mitochondria through a deficit in mitochondrial transcription factor A (TFAM) can propel the aging process. This untreated metabolic failure in T cells leads to the build up in circulating cytokines, resulting in a chronic inflammatory state reminiscent of “inflammaging,” a hallmark of aging. The resulting cytokine storm serves as a systemic trigger that drives the aging process [94]. The relationship between cellular energy production impairment and inflammation is the fact that mitochondrial integrity is essential for maintaining healthy immune system suggests that improving or enhancing.

Moreover, mitochondria are essential for energy production, supporting aerobic respiration, and various cellular processes including differentiation, signal transmission, and apoptosis. Nicotinamide adenine dinucleotide (NAD + ), vital for mitochondrial energy generation, declines as organisms age. New findings have proposed a novel approach to enhance the generation of NAD+ by blocking the activity of 2-amino-3-carboxylic muconic acid 6-hemaldehyde decarboxylase (n). This enhancement in NAD+ levels has been demonstrated to bolster mitochondrial functionality in both Caenorhabditis elegans and mice [95]. This research suggests that promoting NAD+ production can help restore mitochondrial function, which is critical for maintaining cellular energy metabolism and overall cellular health, especially in aging or dysfunctional cells.

In addition to targeting traditional metabolic pathways, emerging therapies such as NAD+ precursors and mitochondrial transplantation hold promise as novel interventions for reversing T cell senescence. NAD+ precursors, such as nicotinamide riboside (NR) and nicotinamide mononucleotide (NMN), have garnered significant attention due to their ability to restore NAD+ levels and improve mitochondrial function. For example, research has found that T cells from B16F10 tumor-bearing mice exhibit aging characteristics and mitochondrial dysfunction. Treatment with NMN enhances mitochondrial function by increasing NAD+ levels, reducing mitochondrial DNA (mtDNA) in the cytoplasm, thereby alleviating STING-mediated T cell senescence [96]. However, the clinical application of NAD+ precursors is still under investigation. While animal studies have demonstrated the benefits of NMN and NR, the long-term safety and efficacy of these supplements in humans remain uncertain. Concerns also exist regarding dose-dependent effects, as excessively high NAD+ levels may inadvertently alter gene expression and interfere with other metabolic pathways.

Another emerging therapeutic strategy is mitochondrial transplantation, which involves the transfer of healthy mitochondria into damaged or aging cells. This approach has shown promising results in preclinical models for rejuvenating aging cells and restoring their function [97]. A recent study discovered that mitochondrial transplantation can promote protective effector and memory CD4+ T cell responses during Mycobacterium tuberculosis infection, while simultaneously diminishing exhaustion and senescence in elderly CD4+ T cells. This research highlights the potential of mitochondrial transplantation as a therapeutic strategy to rejuvenate aging immune cells and restore their functionality [98]. Although this strategy holds potential, it faces several clinical challenges, including the risk of immune rejection, the cost of mitochondria isolation and transplantation, and the technical complexity of delivering mitochondria effectively. Moreover, safety concerns regarding the long-term effects of mitochondrial transplantation, such as potential tumorigenesis or off-target effects, need to be addressed before this approach can be applied clinically [99].

### Combined immune checkpoint inhibitor therapy

Immunotherapy uses immune checkpoint inhibitors (ICIs) to treat melanoma, lung cancer, and other cancers. Anti-PD-1/PD-L1 monoclonal antibodies have been developed as ICIs to target immune evasion by tumors [100]. Anti-PD-1/PD-L1 monoclonal antibodies bind to PD-1 and PD-L1 receptors on T cells and tumor cells, respectively, disrupting the PD-1/PD-L1 interaction and reactivating anti-tumor T cell responses. The anti-tumor effect of ICIs is linked to T cell activation and CD28 expression; hence, this therapy is effective against exhausted T cells but not against senescent T cells [101].

However, research indicates that preventing tumor-specific T cell senescence via ATM and/or MAPK signaling inhibition combined with anti-PD-L1 checkpoint blockade can synergistically enhance anti-tumor immunity and immunotherapy efficacy in vivo [102]. Moreover, melanoma and colon tumor growth were inhibited in mice treated with nicotinamide riboside and an anti-PD-1 antibody. The combination of nicotinamide riboside supplementation and ICI therapy produced additive anti-tumor effects. These findings suggest that the removal of senescent T cells is a crucial factor in the success of cancer immunotherapy [103].

## Concluding remarks and future perspectives

The exploration of metabolic reprogramming in senescent T cells is not just a promising avenue for cancer therapy; it heralds a paradigm shift in how we understand and treat the interplay between aging and immune competence. As researchers delve deeper into the metabolic underpinnings of T cell senescence, they uncover potential strategies to reverse senescence or harness its mechanisms to bolster anti-tumor immunity. This emerging branch provides new outlooks to improve the effectiveness of the treatment of cancer using immunotherapy and developing the approaches to modulate aging immune system.

Further studies needed to be directed on the applied aspect of these discoveries. In particular, clinical trials investigating metabolic modulators aimed at reversing the senescent T cell phenotype could provide the necessary evidence for translating these findings from basic research to clinical practice. For instance, the role of drugs like rapamycin, which has shown promise in enhancing immune functions by modulating mTOR pathways, could be further explored in larger patient cohorts to assess its benefits and limitations in a clinical setting [104]. Additionally, clinical trials focused on metabolic therapies targeting key pathways such as glycolysis and lipid metabolism are crucial to assess their therapeutic effects on reversing T cell senescence and improving anti-tumor responses.

Moreover, understanding the specific metabolic pathways that contribute to T cell senescence and tumor resistance could open up new therapeutic avenues. For example, targeting glycolysis and lipid metabolism in T cells has been suggested to improve their proliferation and functionality [105]. Integrating these metabolic therapies with existing treatments, such as checkpoint inhibitors, could potentially overcome some of the current limitations of cancer immunotherapies. This combination could be tested in clinical trials to assess synergistic effects and optimize cancer immunotherapy regimens.

Lastly, there is an essential discussion regarding the mutual crosstalk between senescent T cells and other components of the tumor milieu. Isolated analysis has revealed that characteristics of the TME can significantly affect the metabolic and functional status of infiltrating immune cells [106]. It is possible to suppose that interrupting these pathological interactions and affecting certain metabolic processes in the tumor environment can reverse T cell exhaustion and enhance their therapeutic efficacy. Clinical trials investigating the impact of TME-targeted therapies in combination with metabolic modulation could pave the way for novel therapeutic strategies. Additionally, by regulating metabolic changes controlled by epigenetic modifications, it may be possible to delay T cell aging, enhance their function, and thereby boost immune responses. Specific epigenetic and metabolic interventions could help restore the function of aging T cells, improve immune responses in the elderly, and reduce the risk of infections and diseases. However, targeting epigenetic and metabolic pathways may lead to widespread changes in gene expression, potentially causing unintended side effects or affecting other cell types [107]. Moreover, individual differences in epigenetic and metabolic states may impact the uniformity and predictability of treatment outcomes [108].

Also, the use of other newly-developed technologies like single-cell RNA sequencing could shed the light on relative heterogeneity of the T cell senescing and its metabolism regulation [109]. Such technologies can help identify new biomarkers of senescence and pinpoint targetable metabolic vulnerabilities within individual T cells. These innovations could facilitate the identification of patients most likely to benefit from metabolic-based therapies and optimize personalized treatment approaches.

Therefore, understanding metabolic reprogramming of senescent T cells in cancer allows considering further the immune-based therapies’ extension and enhancing the patients’ quality of life in cancer. As we advance, the interplay of metabolism, aging, and cancer will undoubtedly reveal even more about how we can harness the body’s own mechanisms to fight disease and decay. Future clinical trials focusing on metabolic modulation and epigenetic interventions will be crucial for translating these findings into effective cancer therapies.

## References

[1] Quinn KM, Vicencio DM, La Gruta NL. The paradox of aging: aging-related shifts in T cell function and metabolism. Semin Immunol. 2023;70:101834. 37659169 10.1016/j.smim.2023.101834

[2] Kasamatsu T. Implications of senescent T cells for cancer immunotherapy. Cancers. 2023;15:5835. 38136380 10.3390/cancers15245835 PMC10742305

[3] Huang M, Wang Y, Fang L, Liu C, Feng F, Liu L, et al. T cell senescence: a new perspective on immunotherapy in lung cancer. Front Immunol. 2024;15:1338680. 38415245 10.3389/fimmu.2024.1338680 PMC10896971

[4] Matveeva K, Vasilieva M, Minskaia E, Rybtsov S, Shevyrev D. T-cell immunity against senescence: potential role and perspectives. Front Immunol. 2024;15:1360109. 38504990 10.3389/fimmu.2024.1360109 PMC10948549

[5] Chebly A, Khalil C, Kuzyk A, Beylot-Barry M, Chevret E. T-cell lymphocytes’ aging clock: telomeres, telomerase and aging. Biogerontology. 2024;25:279–88. 37917220 10.1007/s10522-023-10075-6

[6] Gressler AE, Leng H, Zinecker H, Simon AK. Proteostasis in T cell aging. Semin Immunol. 2023;70:101838. 37708826 10.1016/j.smim.2023.101838 PMC10804938

[7] Liu Z, Liang Q, Ren Y, Guo C, Ge X, Wang L, et al. Immunosenescence: molecular mechanisms and diseases. Signal Transduct Target Ther. 2023;8:200. 37179335 10.1038/s41392-023-01451-2 PMC10182360

[8] Akbar AN, Henson SM. Are senescence and exhaustion intertwined or unrelated processes that compromise immunity? Nat Rev Immunol. 2011;11:289–95. 21436838 10.1038/nri2959

[9] Ye J, Huang X, Hsueh EC, Zhang Q, Ma C, Zhang Y, et al. Human regulatory T cells induce T-lymphocyte senescence. Blood. 2012;120:2021–31. 22723548 10.1182/blood-2012-03-416040 PMC3437594

[10] Mondal AM, Horikawa I, Pine SR, Fujita K, Morgan KM, Vera E, et al. p53 isoforms regulate aging- and tumor-associated replicative senescence in T lymphocytes. J Clin Invest. 2013;123:5247–57. 24231352 10.1172/JCI70355 PMC3859419

[11] Zhao Y, Shao Q, Peng G. Exhaustion and senescence: two crucial dysfunctional states of T cells in the tumor microenvironment. Cell Mol Immunol. 2020;17:27–35. 31853000 10.1038/s41423-019-0344-8 PMC6952436

[12] Akbar AN, Henson SM, Lanna A. Senescence of T lymphocytes: implications for enhancing human immunity. Trends Immunol. 2016;37:866–76. 27720177 10.1016/j.it.2016.09.002

[13] Ahmed R, Miners KL, Lahoz-Beneytez J, Jones RE, Roger L, Baboonian C, et al. CD57(+) memory T cells proliferate in vivo. Cell Rep. 2020;33:108501. 33326780 10.1016/j.celrep.2020.108501 PMC7758161

[14] Czesnikiewicz-Guzik M, Lee WW, Cui D, Hiruma Y, Lamar DL, Yang ZZ, et al. T cell subset-specific susceptibility to aging. Clin Immunol. 2008;127:107–18. 18222733 10.1016/j.clim.2007.12.002 PMC2435295

[15] Callender LA, Carroll EC, Bober EA, Akbar AN, Solito E, Henson SM. Mitochondrial mass governs the extent of human T cell senescence. Aging Cell. 2020;19:e13067. 31788930 10.1111/acel.13067 PMC6996952

[16] Pereira BI, De Maeyer RPH, Covre LP, Nehar-Belaid D, Lanna A, Ward S, et al. Sestrins induce natural killer function in senescent-like CD8(+) T cells. Nat Immunol. 2020;21:684–94. 32231301 10.1038/s41590-020-0643-3 PMC10249464

[17] Mogilenko DA, Shpynov O, Andhey PS, Arthur L, Swain A, Esaulova E, et al. Comprehensive profiling of an aging immune system reveals clonal GZMK(+) CD8(+) T cells as conserved hallmark of inflammaging. Immunity. 2021;54:99–115.e112. 33271118 10.1016/j.immuni.2020.11.005

[18] Cao J, Liao S, Zeng F, Liao Q, Luo G, Zhou Y. Effects of altered glycolysis levels on CD8(+) T cell activation and function. Cell Death Dis. 2023;14:407. 37422501 10.1038/s41419-023-05937-3 PMC10329707

[19] Jeng MY, Hull PA, Fei M, Kwon HS, Tsou CL, Kasler H, et al. Metabolic reprogramming of human CD8(+) memory T cells through loss of SIRT1. J Exp Med. 2018;215:51–62. 29191913 10.1084/jem.20161066 PMC5748845

[20] Lanna A, Gomes DC, Muller-Durovic B, McDonnell T, Escors D, Gilroy DW, et al. A sestrin-dependent Erk-Jnk-p38 MAPK activation complex inhibits immunity during aging. Nat Immunol. 2017;18:354–63. 28114291 10.1038/ni.3665 PMC5321575

[21] Davenport B, Eberlein J, van der Heide V, Jhun K, Nguyen TT, Victorino F, et al. Aging of antiviral CD8(+) memory T cells fosters increased survival, metabolic adaptations, and lymphoid tissue homing. J Immunol. 2019;202:460–75. 30552164 10.4049/jimmunol.1801277 PMC6358025

[22] Liu X, Hartman CL, Li L, Albert CJ, Si F, Gao A, et al. Reprogramming lipid metabolism preventseffector T cell senescence and enhances tumor immunotherapy. Sci Transl Med. 2021;13:eaaz6314. 33790024 10.1126/scitranslmed.aaz6314 PMC12040281

[23] Dozmorov MG, Coit P, Maksimowicz-McKinnon K, Sawalha AH. Age-associated DNA methylation changes in naive CD4(+) T cells suggest an evolving autoimmune epigenotype in aging T cells. Epigenomics. 2017;9:429–45. 28322571 10.2217/epi-2016-0143 PMC5549647

[24] Lanna A, Henson SM, Escors D, Akbar AN. The kinase p38 activated by the metabolic regulator AMPK and scaffold TAB1 drives the senescence of human T cells. Nat Immunol. 2014;15:965–72. 25151490 10.1038/ni.2981 PMC4190666

[25] Callender LA, Carroll EC, Beal RWJ, Chambers ES, Nourshargh S, Akbar AN, et al. Human CD8(+) EMRA T cells display a senescence-associated secretory phenotype regulated by p38 MAPK. Aging Cell. 2018;17:e12675. 29024417 10.1111/acel.12675 PMC5770853

[26] Lucas CL, Kuehn HS, Zhao F, Niemela JE, Deenick EK, Palendira U, et al. Dominant-activating germline mutations in the gene encoding the PI(3)K catalytic subunit p110delta result in T cell senescence and human immunodeficiency. Nat Immunol. 2014;15:88–97. 24165795 10.1038/ni.2771 PMC4209962

[27] Kim C, Hu B, Jadhav RR, Jin J, Zhang H, Cavanagh MM, et al. Activation of miR-21-regulated pathways in immune aging selects against signatures characteristic of memory T cells. Cell Rep. 2018;25:2148–62.e2145. 30463012 10.1016/j.celrep.2018.10.074 PMC6371971

[28] Li L, Liu X, Sanders KL, Edwards JL, Ye J, Si F, et al. TLR8-mediated metabolic control of human treg function: a mechanistic target for cancer immunotherapy. Cell Metab. 2019;29:103–123.e105. 30344014 10.1016/j.cmet.2018.09.020 PMC7050437

[29] Guo Z, Wang G, Wu B, Chou WC, Cheng L, Zhou C, et al. DCAF1 regulates Treg senescence via the ROS axis during immunological aging. J Clin Invest. 2020;130:5893–908. 32730228 10.1172/JCI136466 PMC7598062

[30] Liu Z, Ning F, Cai Y, Sheng H, Zheng R, Yin X, et al. The EGFR-P38 MAPK axis up-regulates PD-L1 through miR-675-5p and down-regulates HLA-ABC via hexokinase-2 in hepatocellular carcinoma cells. Cancer Commun. 2021;41:62–78. 10.1002/cac2.12117 PMC7819566 34236149

[31] Liu QP, Luo Q, Deng B, Ju Y, Song GB. Stiffer matrix accelerates migration of hepatocellular carcinoma cells through enhanced aerobic glycolysis via the MAPK-YAP signaling. Cancers. Cancers. 2020;12:490. 32093118 10.3390/cancers12020490 PMC7072284

[32] Janelle V, Neault M, Lebel ME, De Sousa DM, Boulet S, Durrieu L, et al. p16(INK4a) regulates cellular senescence in PD-1-expressing human T cells. Front Immunol. 2021;12:698565. 34434190 10.3389/fimmu.2021.698565 PMC8381277

[33] Truong TP, Sakata-Yanagimoto M, Yamada M, Nagae G, Enami T, Nakamoto-Matsubara R, et al. Age-dependent decrease of DNA hydroxymethylation in human T cells. J Clin Exp Hematop. 2015;55:1–6. 26105999 10.3960/jslrt.55.1

[34] Kaeberlein M, McVey M, Guarente L. The SIR2/3/4 complex and SIR2 alone promote longevity in *Saccharomyces cerevisiae* by two different mechanisms. Genes Dev. 1999;13:2570–80. 10521401 10.1101/gad.13.19.2570 PMC317077

[35] Tissenbaum HA, Guarente L. Increased dosage of a sir-2 gene extends lifespan in *Caenorhabditis elegans*. Nature. 2001;410:227–30. 11242085 10.1038/35065638

[36] Zhou Y, Li GY, Ren JP, Wang L, Zhao J, Ning SB, et al. Protection of CD4+ T cells from hepatitis C virus infection-associated senescence via DeltaNp63-miR-181a-Sirt1 pathway. J Leukoc Biol. 2016;100:1201–11. 27354409 10.1189/jlb.5A0316-119RR PMC5069086

[37] Cheng T, Ding S, Liu S, Li Y, Sun L. Human umbilical cord-derived mesenchymal stem cell therapy ameliorates lupus through increasing CD4+ T cell senescence via MiR-199a-5p/Sirt1/p53 axis. Theranostics. 2021;11:893–905. 33391511 10.7150/thno.48080 PMC7738872

[38] Wang Y, Bi Y, Chen X, Li C, Li Y, Zhang Z, et al. Histone deacetylase SIRT1 negatively regulates the differentiation of interleukin-9-producing CD4(+) T cells. Immunity. 2016;44:1337–49. 27317260 10.1016/j.immuni.2016.05.009

[39] Wilfahrt D, Delgoffe GM. Metabolic waypoints during T cell differentiation. Nat Immunol. 2024;25:206–17. 38238609 10.1038/s41590-023-01733-5

[40] Nicoli F, Cabral-Piccin MP, Papagno L, Gallerani E, Fusaro M, Folcher V, et al. Altered basal lipid metabolism underlies the functional impairment of naive CD8(+) T cells in elderly humans. J Immunol. 2022;208:562–70. 35031578 10.4049/jimmunol.2100194 PMC7615155

[41] Callender LA, Carroll EC, Garrod-Ketchley C, Schroth J, Bystrom J, Berryman V, et al. Altered nutrient uptake causes mitochondrial dysfunction in senescent CD8(+) EMRA T Cells During Type 2 diabetes. Front Aging. 2021;2:681428. 35821991 10.3389/fragi.2021.681428 PMC9261431

[42] Larbi A, Fortin C, Dupuis G, Berrougui H, Khalil A, Fulop T. Immunomodulatory role of high-density lipoproteins: impact on immunosenescence. Age. 2014;36:9712. 25216565 10.1007/s11357-014-9712-6 PMC4162887

[43] Bazioti V, La Rose AM, Maassen S, Bianchi F, de Boer R, Halmos B, et al. T cell cholesterol efflux suppresses apoptosis and senescence and increases atherosclerosis in middle aged mice. Nat Commun. 2022;13:3799. 35778407 10.1038/s41467-022-31135-4 PMC9249754

[44] Cheng X, Tan X, Wang W, Zhang Z, Zhu R, Wu M, et al. Long-Chain Acylcarnitines Induce Senescence of Invariant Natural Killer T Cells in Hepatocellular Carcinoma. Cancer Res. 2023;83:582–94. 36512635 10.1158/0008-5472.CAN-22-2273

[45] Gao A, Liu X, Lin W, Wang J, Wang S, Si F. et al. Tumor-derived ILT4 induces T cell senescence andsuppresses tumor immunity. J Immunother Cancer. 2021;9:e001536. 33653799 10.1136/jitc-2020-001536 PMC7929805

[46] Hu X, Yasuda T, Yasuda-Yosihara N, Yonemura A, Umemoto T, Nakachi Y, et al. Downregulation of 15-PGDH enhances MASH-HCC development via fatty acid-induced T-cell exhaustion. JHEP Rep. 2023;5:100892. 37942226 10.1016/j.jhepr.2023.100892 PMC10628853

[47] Vaena S, Chakraborty P, Lee HG, Janneh AH, Kassir MF, Beeson G, et al. Aging-dependent mitochondrial dysfunction mediated by ceramide signaling inhibits antitumor T cell response. Cell Rep. 2021;35:109076. 33951438 10.1016/j.celrep.2021.109076 PMC8127241

[48] Park JW, Park WJ, Futerman AH. Ceramide synthases as potential targets for therapeutic intervention in human diseases. Biochim Biophys Acta. 2014;1841:671–81. 24021978 10.1016/j.bbalip.2013.08.019

[49] Ma M, Yang Y, Chen Z, Li X, Yang Z, Wang K, et al. T-cell senescence induced by peripheral phospholipids. Cell Biol Toxicol. 2023;39:2937–52. 37261679 10.1007/s10565-023-09811-y

[50] Ernster L, Schatz G. Mitochondria: a historical review. J Cell Biol. 1981;91:227s–255s. 7033239 10.1083/jcb.91.3.227s PMC2112799

[51] Finkel T. Signal transduction by reactive oxygen species. J Cell Biol. 2011;194:7–15. 21746850 10.1083/jcb.201102095 PMC3135394

[52] Oberst A, Bender C, Green DR. Living with death: the evolution of the mitochondrial pathway of apoptosis in animals. Cell Death Differ. 2008;15:1139–46. 18451868 10.1038/cdd.2008.65 PMC2612587

[53] Miwa S, Kashyap S, Chini E, von Zglinicki T. Mitochondrial dysfunction in cell senescence and aging. J Clin Investig. 2022;132:e158447. 35775483 10.1172/JCI158447 PMC9246372

[54] Miwa S, Jow H, Baty K, Johnson A, Czapiewski R, Saretzki G, et al. Low abundance of the matrix arm of complex I in mitochondria predicts longevity in mice. Nat Commun. 2014;5:3837. 24815183 10.1038/ncomms4837 PMC4024759

[55] Dencher NA, Frenzel M, Reifschneider NH, Sugawa M, Krause F. Proteome alterations in rat mitochondria caused by aging. Ann N Y Acad Sci. 2007;1100:291–8. 17460190 10.1196/annals.1395.030

[56] Boengler K, Kosiol M, Mayr M, Schulz R, Rohrbach S. Mitochondria and ageing: role in heart, skeletal muscle and adipose tissue. J Cachexia Sarcopenia Muscle. 2017;8:349–69. 28432755 10.1002/jcsm.12178 PMC5476857

[57] Drouet M, Lauthier F, Charmes JP, Sauvage P, Ratinaud MH. Age-associated changes in mitochondrial parameters on peripheral human lymphocytes. Exp Gerontol. 1999;34:843–52. 10622419 10.1016/s0531-5565(99)00058-3

[58] Henson SM, Lanna A, Riddell NE, Franzese O, Macaulay R, Griffiths SJ, et al. p38 signaling inhibits mTORC1-independent autophagy in senescent human CD8(+) T cells. J Clin Invest. 2014;124:4004–16. 25083993 10.1172/JCI75051 PMC4151208

[59] Siska PJ, Beckermann KE, Mason FM, Andrejeva G, Greenplate AR, Sendor AB. et al. Mitochondrial dysregulation and glycolytic insufficiency functionally impair CD8 T cells infiltrating human renal cell carcinoma. JCI Insight. 2017;2:e93411. 28614802 10.1172/jci.insight.93411 PMC5470888

[60] Xiao L, Ma X, Ye L, Su P, Xiong W, Bi E, et al. IL-9/STAT3/fatty acid oxidation-mediated lipid peroxidation contributes to Tc9 cell longevity and enhanced antitumor activity. J Clin Invest. 2022;132:e153247. 10.1172/JCI153247 PMC8970676 35192544

[61] Schank M, Zhao J, Wang L, Nguyen LNT, Zhang Y, Wu XY. et al. ROS-induced mitochondrial dysfunction in CD4 T cells from ART-controlled people living with HIV. Viruses. 2023;15:1061. 37243148 10.3390/v15051061 PMC10224005

[62] Ferree A, Shirihai O. Mitochondrial dynamics: the intersection of form and function. Adv Exp Med Biol. 2012;748:13–40. 22729853 10.1007/978-1-4614-3573-0_2 PMC5967395

[63] Sebastian D, Palacin M, Zorzano A. Mitochondrial dynamics: coupling mitochondrial fitness with healthy aging. Trends Mol Med. 2017;23:201–15. 28188102 10.1016/j.molmed.2017.01.003

[64] Liu YJ, McIntyre RL, Janssens GE, Houtkooper RH. Mitochondrial fission and fusion: a dynamic role in aging and potential target for age-related disease. Mech Ageing Dev. 2020;186:111212. 32017944 10.1016/j.mad.2020.111212

[65] Smirnova E, Griparic L, Shurland DL, van der Bliek AM. Dynamin-related protein Drp1 is required for mitochondrial division in mammalian cells. Mol Biol Cell. 2001;12:2245–56. 11514614 10.1091/mbc.12.8.2245 PMC58592

[66] Yu T, Robotham JL, Yoon Y. Increased production of reactive oxygen species in hyperglycemic conditions requires dynamic change of mitochondrial morphology. Proc Natl Acad Sci USA. 2006;103:2653–8. 16477035 10.1073/pnas.0511154103 PMC1413838

[67] Taguchi N, Ishihara N, Jofuku A, Oka T, Mihara K. Mitotic phosphorylation of dynamin-related GTPase Drp1 participates in mitochondrial fission. J Biol Chem. 2007;282:11521–9. 17301055 10.1074/jbc.M607279200

[68] Youle RJ, Karbowski M. Mitochondrial fission in apoptosis. Nat Rev Mol Cell Biol. 2005;6:657–63. 16025099 10.1038/nrm1697

[69] Cipolat S, Martins de Brito O, Dal Zilio B, Scorrano L. OPA1 requires mitofusin 1 to promote mitochondrial fusion. Proc Natl Acad Sci USA. 2004;101:15927–32. 15509649 10.1073/pnas.0407043101 PMC528769

[70] Sharma A, Smith HJ, Yao P, Mair WB. Causal roles of mitochondrial dynamics in longevity and healthy aging. EMBO Rep. 2019;20:e48395. 31667999 10.15252/embr.201948395 PMC6893295

[71] Cogliati S, Frezza C, Soriano ME, Varanita T, Quintana-Cabrera R, Corrado M, et al. Mitochondrial cristae shape determines respiratory chain supercomplexes assembly and respiratory efficiency. Cell. 2013;155:160–71. 24055366 10.1016/j.cell.2013.08.032 PMC3790458

[72] de Brito OM, Scorrano L. Mitofusin 2 tethers endoplasmic reticulum to mitochondria. Nature. 2008;456:605–10. 19052620 10.1038/nature07534

[73] Zukowski E, Sannella M, Rockhold JD, Kalantar GH, Yu J, SantaCruz-Calvo S, et al. STAT3 modulates CD4(+) T mitochondrial dynamics and function in aging. Aging Cell. 2023;22:e13996. 37837188 10.1111/acel.13996 PMC10652300

[74] Passos JF, Nelson G, Wang C, Richter T, Simillion C, Proctor CJ, et al. Feedback between p21 and reactive oxygen production is necessary for cell senescence. Mol Syst Biol. 2010;6:347. 20160708 10.1038/msb.2010.5 PMC2835567

[75] Davis T, Bagley MC, Dix MC, Murziani PG, Rokicki MJ, Widdowson CS, et al. Synthesis and in vivo activity of MK2 and MK2 substrate-selective p38alpha(MAPK) inhibitors in Werner syndrome cells. Bioorg Med Chem Lett. 2007;17:6832–5. 17964780 10.1016/j.bmcl.2007.10.036

[76] Mannick JB, Del Giudice G, Lattanzi M, Valiante NM, Praestgaard J, Huang B, et al. mTOR inhibition improves immune function in the elderly. Sci Transl Med. 2014;6:268ra179. 25540326 10.1126/scitranslmed.3009892

[77] Schettini F, Sobhani N, Ianza A, Triulzi T, Molteni A, Lazzari MC, et al. Immune system and angiogenesis-related potential surrogate biomarkers of response to everolimus-based treatment in hormone receptor-positive breast cancer: an exploratory study. Breast Cancer Res Treat. 2020;184:421–31. 32770287 10.1007/s10549-020-05856-3 PMC7599144

[78] Templeton AJ, Dutoit V, Cathomas R, Rothermundt C, Bartschi D, Droge C, et al. Phase 2 trial of single-agent everolimus in chemotherapy-naive patients with castration-resistant prostate cancer (SAKK 08/08). Eur Urol. 2013;64:150–8. 23582881 10.1016/j.eururo.2013.03.040

[79] Zhang Y, Yan H, Xu Z, Yang B, Luo P, He Q. Molecular basis for class side effects associated with PI3K/AKT/mTOR pathway inhibitors. Expert Opin Drug Metab Toxicol. 2019;15:767–74. 31478386 10.1080/17425255.2019.1663169

[80] Fan C, Wunderlich M, Cai X, Yan Z, Zhang F, Davis AK, et al. Kinase-independent role of mTOR and on-/off-target effects of an mTOR kinase inhibitor. Leukemia. 2023;37:2073–81. 37532788 10.1038/s41375-023-01987-w

[81] Battram AM, Bachiller M, Martin-Antonio B. Senescence in the development and response to cancer with immunotherapy: a double-edged sword. Int J Mol Sci. 2020;21:4346. 32570952 10.3390/ijms21124346 PMC7352478

[82] Wang Q, Qin Y, Li B. CD8(+) T cell exhaustion and cancer immunotherapy. Cancer Lett. 2023;559:216043. 36584935 10.1016/j.canlet.2022.216043

[83] Titov A, Kaminskiy Y, Ganeeva I, Zmievskaya E, Valiullina A, Rakhmatullina A. et al. Knowns and unknowns about CAR-T cell dysfunction. Cancers. 2022;14:1078. 35205827 10.3390/cancers14041078 PMC8870103

[84] Johnson DB, Sosman JA. Update on the targeted therapy of melanoma. Curr Treat Options Oncol. 2013;14:280–92. 23420410 10.1007/s11864-013-0226-8 PMC6684217

[85] Manic G, Obrist F, Sistigu A, Vitale I. Trial watch: targeting ATM-CHK2 and ATR-CHK1 pathways for anticancer therapy. Mol Cell Oncol. 2015;2:e1012976. 27308506 10.1080/23723556.2015.1012976 PMC4905354

[86] Awasthi A, Raju MB, Rahman MA. Current insights of inhibitors of p38 mitogen-activated protein kinase in inflammation. Med Chem. 2021;17:555–75. 32106802 10.2174/1573406416666200227122849

[87] Zhang J, He T, Xue L, Guo H. Senescent T cells: a potential biomarker and target for cancer therapy. EBioMedicine. 2021;68:103409. 34049248 10.1016/j.ebiom.2021.103409 PMC8170103

[88] Ye J, Ma C, Hsueh EC, Dou J, Mo W, Liu S, et al. TLR8 signaling enhances tumor immunity by preventing tumor-induced T-cell senescence. EMBO Mol Med. 2014;6:1294–311. 25231413 10.15252/emmm.201403918 PMC4287933

[89] Sohrabi S, Masoumi J, Naseri B, Ghorbaninezhad F, Alipour S, Kazemi T, et al. STATs signaling pathways in dendritic cells: As potential therapeutic targets? Int Rev Immunol. 2024;43:138–59. 37886903 10.1080/08830185.2023.2274576

[90] Desnues B, Macedo AB, Roussel-Queval A, Bonnardel J, Henri S, Demaria O, et al. TLR8 on dendritic cells and TLR9 on B cells restrain TLR7-mediated spontaneous autoimmunity in C57BL/6 mice. Proc Natl Acad Sci USA. 2014;111:1497–502. 24474776 10.1073/pnas.1314121111 PMC3910605

[91] Vargas-Hernandez A, Forbes LR. JAK/STAT proteins and their biological impact on NK cell development and function. Mol Immunol. 2019;115:21–30. 30704805 10.1016/j.molimm.2018.12.005

[92] Veneziani I, Alicata C, Moretta L, Maggi E. Human toll-like receptor 8 (TLR8) in NK cells: Implication for cancer immunotherapy. Immunol Lett. 2023;261:13–16. 37451320 10.1016/j.imlet.2023.07.003

[93] Jing JL, Ning TCY, Natali F, Eisenhaber F, Alfatah M. Iron supplementation delays aging and extends cellular lifespan through potentiation of mitochondrial function. Cells. 2022;11:862. 35269484 10.3390/cells11050862 PMC8909192

[94] Desdin-Mico G, Soto-Heredero G, Aranda JF, Oller J, Carrasco E, Gabande-Rodriguez E, et al. T cells with dysfunctional mitochondria induce multimorbidity and premature senescence. Science. 2020;368:1371–6. 32439659 10.1126/science.aax0860 PMC7616968

[95] Katsyuba E, Mottis A, Zietak M, De Franco F, van der Velpen V, Gariani K, et al. De novo NAD(+) synthesis enhances mitochondrial function and improves health. Nature. 2018;563:354–9. 30356218 10.1038/s41586-018-0645-6 PMC6448761

[96] Ye B, Pei Y, Wang L, Meng D, Zhang Y, Zou S, et al. NAD(+) supplementation prevents STING-induced senescence in CD8(+) T cells by improving mitochondrial homeostasis. J Cell Biochem. 2024;125:e30522. 38224175 10.1002/jcb.30522

[97] Zhao R, Dong C, Liang Q, Gao J, Sun C, Gu Z, et al. Engineered mitochondrial transplantation as an anti-aging therapy. Aging Dis. 2024; 10.14336/AD.2024.0231. 10.14336/AD.2024.0231 PMC12221395 39122452

[98] Headley CA, Gautam S, Olmo-Fontanez A, Garcia-Vilanova A, Dwivedi V, Schami A, et al. Mitochondrial transplantation promotes protective effector and memory CD4(+) T cell response during mycobacterium tuberculosis infection and diminishes exhaustion and senescence in elderly CD4(+) T cells. Adv Sci. 2024;11:e2401077. 10.1002/advs.202401077 PMC11423092 39039808

[99] Yamada Y, Ito M, Arai M, Hibino M, Tsujioka T, Harashima H. Challenges in promoting mitochondrial transplantation therapy. Int J Mol Sci. 2020;21:6365. 32887310 10.3390/ijms21176365 PMC7504154

[100] Cha JH, Chan LC, Li CW, Hsu JL, Hung MC. Mechanisms controlling PD-L1 Expression in Cancer. Mol Cell. 2019;76:359–70. 31668929 10.1016/j.molcel.2019.09.030 PMC6981282

[101] Kamphorst AO, Wieland A, Nasti T, Yang S, Zhang R, Barber DL, et al. Rescue of exhausted CD8 T cells by PD-1-targeted therapies is CD28-dependent. Science. 2017;355:1423–7. 28280249 10.1126/science.aaf0683 PMC5595217

[102] Liu X, Si F, Bagley D, Ma F, Zhang Y, Tao Y. et al. Blockades of effector T cell senescence and exhaustion synergistically enhance antitumor immunity and immunotherapy. J Immunother Cancer. 2022;10:e005020. 36192086 10.1136/jitc-2022-005020 PMC9535198

[103] Yu YR, Imrichova H, Wang H, Chao T, Xiao Z, Gao M, et al. Disturbed mitochondrial dynamics in CD8(+) TILs reinforce T cell exhaustion. Nat Immunol. 2020;21:1540–51. 33020660 10.1038/s41590-020-0793-3

[104] Asghari F, Karimi MH, Pourfathollah AA. mTORC1 inhibition may improve T lymphocytes affected by aging. Immunopharmacol Immunotoxicol. 2023;45:719–29. 37581412 10.1080/08923973.2023.2232101

[105] Sukumar M, Liu J, Ji Y, Subramanian M, Crompton JG, Yu Z, et al. Inhibiting glycolytic metabolism enhances CD8+ T cell memory and antitumor function. J Clin Invest. 2013;123:4479–88. 24091329 10.1172/JCI69589 PMC3784544

[106] Chang CH, Qiu J, O’Sullivan D, Buck MD, Noguchi T, Curtis JD, et al. Metabolic competition in the tumor microenvironment is a driver of cancer progression. Cell. 2015;162:1229–41. 26321679 10.1016/j.cell.2015.08.016 PMC4864363

[107] Heindel JJ, McAllister KA, Worth L Jr, Tyson FL. Environmental epigenomics, imprinting and disease susceptibility. Epigenetics. 2006;1:1–6. 17998808 10.4161/epi.1.1.2642

[108] Jones PA, Baylin SB. The fundamental role of epigenetic events in cancer. Nat Rev Genet. 2002;3:415–28. 12042769 10.1038/nrg816

[109] Stubbington MJT, Lonnberg T, Proserpio V, Clare S, Speak AO, Dougan G, et al. T cell fate and clonality inference from single-cell transcriptomes. Nat Methods. 2016;13:329–32. 26950746 10.1038/nmeth.3800 PMC4835021

